# An Osteopathic Approach to Foot Drop: A Case Report of Posterior Fibular Head

**DOI:** 10.7759/cureus.72777

**Published:** 2024-10-31

**Authors:** Mikhail Volokitin, Abraham E Libman, Susan Milani, Mary Banihashem, Siam Ayon

**Affiliations:** 1 Osteopathic Manipulative Medicine, Touro College of Osteopathic Medicine, New York, USA; 2 Physical Medicine and Rehabilitation, New York Presbyterian Queens Hospital, New York, USA

**Keywords:** case report, complex regional pain syndrome, foot dorsiflexion, foot drop, high-velocity low-amplitude, muscle energy, nerve compression, nerve dysfunction, osteopathic manipulative treatment, physical rehabilitation

## Abstract

Chronic foot drop can present a diagnostic challenge, often leading to extensive medical evaluations without definitive resolution. We report a case of a 42-year-old female with an elusive cause of foot drop, a complex and engaging case that required extensive medical evaluations. The patient’s complex medical history includes kidney infections, migraine headaches, and irritable bowel syndrome, treated with conventional medications and osteopathic manipulative techniques. The foot drop onset was associated with a gym injury, compounded by prior inconspicuous anatomical variations revealed by imaging. Despite exhaustive neurological evaluations, traditional treatments, and alternative medicine approaches, the etiology remained unclear, and the patient continued to suffer from debilitating symptoms. A notable improvement in foot mobility and associated symptoms was achieved with osteopathic manipulative treatment (OMT), suggesting a possible neuromusculoskeletal link. This case illustrates the diagnostic conundrum of foot drop and highlights OMT as a potential first-line therapy, warranting further investigation into its role in such cases.

## Introduction

Foot drop is diagnosed when a person cannot dorsiflex his/her foot due to weakness or paralysis of the muscles that lift the foot [1]. The most common causes of foot drops are lumbar radiculopathy and peroneal nerve injury [2,3]. Some of the other causes include diabetes, surgery, time spent in a leg cast, muscular disorders, and neurological disorders [4,5]. While imaging can help identify the cause of foot drop, it is often a clinical diagnosis [6]. Current stepwise treatment includes braces, shoe inserts, physical therapy, and, in some cases, surgery [7,8,9].

Osteopathic manipulative treatment (OMT) is a method that is not commonly associated with diagnosing and treating foot drops [10]. It is intriguing to note that multiple database searches have shown only a few case studies that describe a foot drop secondary to common peroneal nerve injury treated successfully with OMT [11]. This rarity adds to the interest and potential of this treatment [1]. Past successes wherein OMT has been used to correct somatic dysfunctions in various body systems made way for the implementation of a comprehensive and individualized treatment regimen. 

We document the case of a 42-year-old female who presented to our osteopathic clinic to manage a long-standing foot drop. Despite trying multiple treatment modalities and interventions, such as physical therapy, acupuncture, and braces, she had not found relief [12,13,14]. She received significant benefits from being treated with OMT and had a complete resolution of her symptoms.

## Case presentation

A 42-year-old caucasian female patient presented with treatment-resistant foot drop, which was managed in our clinic with OMT. Her past medical history included recurrent left-sided kidney infections, migraine headaches, occasional numbness and tingling on the left side of her face, and irritable bowel syndrome (IBS). She had been on synthroid for hypothyroidism for the past 12 years.

Initial symptom onset and incident

The patient began experiencing cramp-like symptoms in her left leg around May 24th, 2022, initially occurring at night and later during the day, which she described as “being unable to move her legs.” On June 7th, 2022, during an aerobic exercise routine at the gym, she twisted her left leg while jumping sideways, resulting in excruciating pain. Despite the pain, she continued exercising briefly before her friend helped her leave the gym due to severe limping and inability to bear weight on her left leg. That night, she noticed paralysis in her left ankle and toes.

Initial medical consultations and treatments

**Figure 1:**
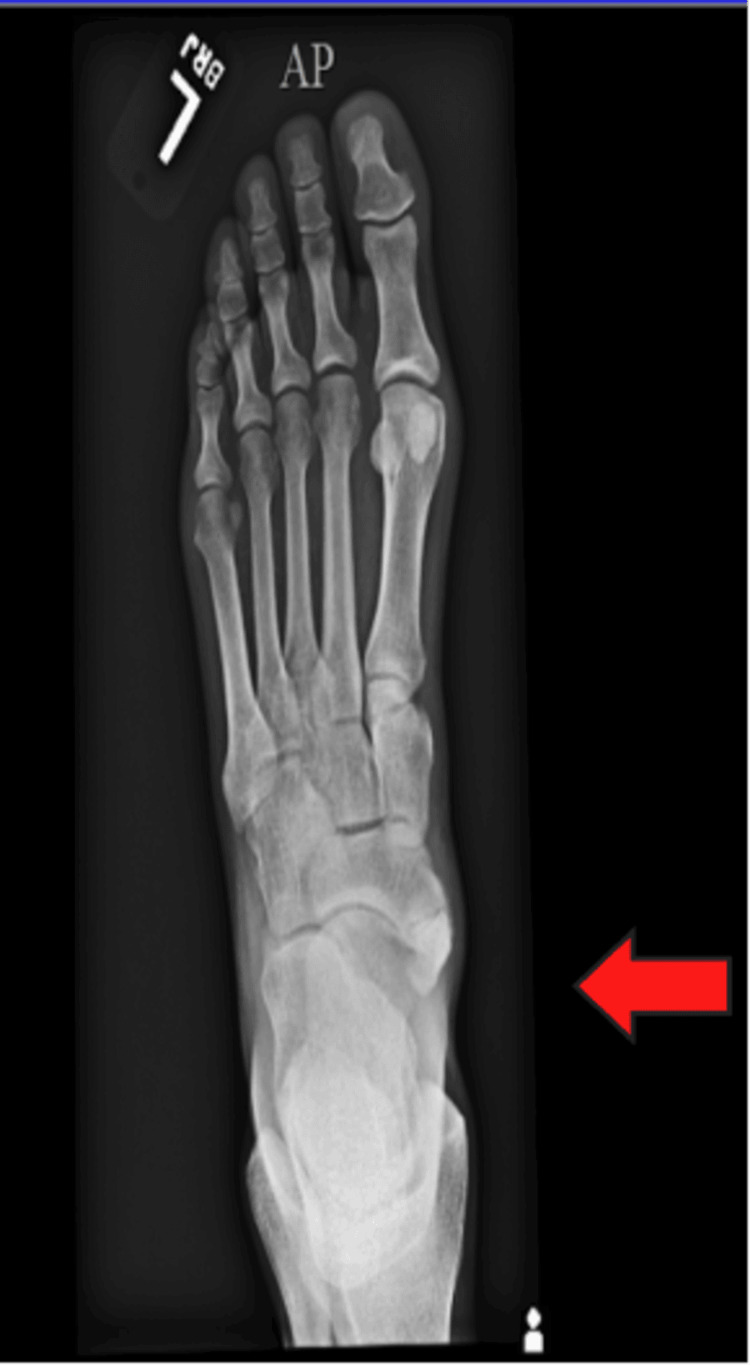
Accessory navicular bone, which may produce posterior tibial tendon irritation/inflammation and inability of single heel raise, all of which were absent in this case on examination.

The patient sought medical care at an urgent care center the next day, where an X-ray suggested a possible broken bone. She was advised to wear a boot for four months. However, an orthopedist later clarified that she had an extra navicular bone, which did not warrant surgery (Figure 1). An MRI revealed some minor partial tears in the ligaments in her left foot. Over the next few months, she consulted multiple orthopedists, neurologists, and a psychiatrist and underwent various MRIs and EMG studies, all yielding inconclusive results.

Neurological and psychiatric evaluations

Despite consultations with several neurologists and a psychiatrist, the patient’s condition remained a mystery. She was suggested to have a psychosomatic cause for her symptoms. She was diagnosed with complex regional pain syndrome (CRPS) by a podiatrist, who prescribed gabapentin and antidepressants, which she did not take. A neurologist prescribed her ketamine cream, which she also did not use.

Path to seeking osteopathic care

After nine months of unsuccessful treatments, including traditional and alternative therapies, the patient was referred to an osteopathic physician. She was diagnosed with left posterior fibular head somatic dysfunction. The left anterior tibialis muscle was atrophic and strained, and the talus was found in the anterior position. The patient received four osteopathic treatments in Spring 2023. These treatments focused on repositioning bones to improve motion and function in the joints of the foot, relieving pressure on the peroneal nerve, and restore compromised blood supply and lymphatic drainage. The techniques employed were mostly direct techniques meant to engage restrictive barriers, including high-velocity low-amplitude (HVLA) to the posterior fibular head and talus, muscle energy for anterior tibialis muscle, direct myofascial release, and pedal pump. The patient reported 75% relief of her symptoms after the first treatment. Due to the previous nine months of persistent pressure from a malpositioned fibular head, the compressed structures, such as lymphatics and blood vessels, required approximately a month for recuperation. Over a period of four months, she experienced significant improvements in leg color, mobility, and overall function, allowing her to travel and return to gym activities.

In September 2023, the patient experienced a relapse while walking in high heels, leading to a recurrence of foot drop symptoms. A notable improvement was once again observed after returning to the same osteopathic physician for two more treatments. She continued to seek further neurological evaluations, including MRIs and Visual Evoked Potential (VEP) tests, all of which were normal. She experienced symptom relief and functional improvements during her osteopathic treatments. This case highlights the diagnostic challenges and complexities of foot drop and the potential benefits of OMT as a symptomatic treatment approach, even in an uncertain underlying etiology.

## Discussion

**Figure 2:**
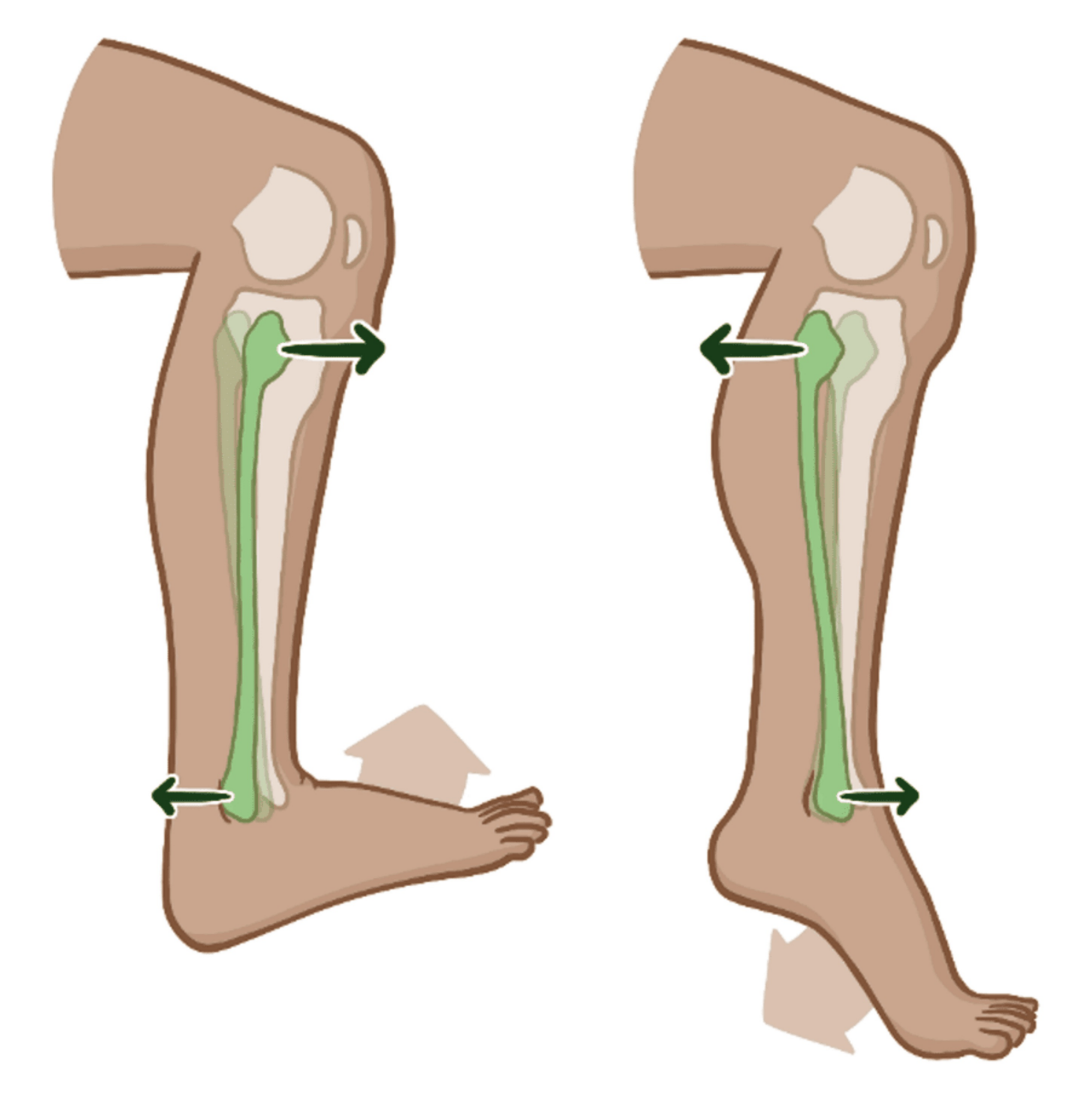
Posterior fibular head somatic dysfunction. Source: [21], Published under the creative commons license.

Foot drop, defined as the inability to dorsiflex the foot due to weakness or paralysis of the dorsiflexor muscles, often due to prior severe supination ankle sprain, poses significant diagnostic and therapeutic challenges. Common etiologies include L5 lumbar radiculopathy secondary to a herniated L4 disc, peroneal nerve entrapment neuropathy at the piriformis (due to piriformis syndrome), or at the fibular head (due to posterior fibular head somatic dysfunction or due to Baker’s cyst) (Figure 2), cauda equina lesion (spinal adhesions), sciatic nerve injury (secondary to injection injury), diabetes, and neurological disorders (lumbosacral plexopathy secondary to colorectal carcinoma) [15]. Standard treatments, such as braces, physical therapy, and surgery, often provide limited relief, particularly in complex cases like the one presented [16,17,18,19].

It is noteworthy that the patient was offered surgery to remove her accessory navicular bone. While an accessory navicular bone may produce posterior tibial tendon irritation/inflammation and the inability to raise a single heel, it cannot lead to posterior fibular head somatic dysfunction. Furthermore, the patient did not have pain around the inside of the ankle and lower leg, so she declined to undergo this procedure.

Osteopathic manipulative treatment

In this case, the patient benefited from osteopathic manipulative treatment (OMT), which involves manual adjustments and manipulations to correct structural imbalances and improve nerve function. The osteopathic approach focused on realigning bones to relieve nerve compression. Techniques including myofascial release, muscle energy, and high-velocity, low-amplitude (HVLA) adjustments were employed to enhance mobility and alleviate pain [5]. The patient reported significant improvement in mobility and a reduction in pain, which greatly improved her quality of life.

The significant symptomatic relief observed in this patient supports the potential of OMT as an effective treatment for foot drop, especially when somatic dysfunctions are present and consistent with biomechanical etiology. This determination aligns with findings from studies on the efficacy of OMT in treating musculoskeletal conditions, demonstrating its versatility and broad applicability in the field of medicine.

Comparison with other treatments

Standard treatments for foot drop include orthotic devices, physical therapy, and surgical interventions. However, these approaches often yield variable results. For instance, orthotic devices can provide support but do not address the underlying cause of nerve compression [7,17,16]. Physical therapy focuses on strengthening and flexibility but may not be sufficient to treat cases of structural misalignment. While potentially effective, surgical options carry risks and may not be suitable for everyone [9,11,12].

In contrast, a study by Tafler et al. (2022) presented a 63-year-old male with chronic left foot drop, which was most likely due to lateral lumbar stenosis and sacroiliac joint dysfunction resulting in radiculopathy [13]. This study highlighted using high-amplitude low-velocity (HALV) techniques to alleviate nerve compression. The patient had been recommended for surgery but experienced significant improvement and resolution of foot drop symptoms following osteopathic treatment. This finding suggests that OMT can serve as an effective alternative to surgical intervention, particularly in cases where the etiology involves neuromusculoskeletal dysfunction. The study delves into the mechanisms by which osteopathic manipulative techniques can relieve nerve compression, reduce pain, and restore function, offering a non-invasive treatment option that addresses the condition’s root cause.

Relevant studies

Studies have shown that OMT can improve function and reduce pain in various musculoskeletal conditions. Altinbilek et al. conducted a single-blind, randomized-controlled trial demonstrating that OMT significantly improved knee osteoarthritis symptoms [1]. This study emphasizes the potential of OMT to manage chronic musculoskeletal pain effectively. Similarly, Tafler et al. reported successful treatment of adhesive capsulitis using osteopathic techniques, reinforcing the applicability of OMT in diverse clinical scenarios [14].

Further supporting the efficacy of OMT, a study by Popovich et al. highlighted the benefits of OMT in treating chronic lower back pain, a condition with similarities to foot drop in terms of nerve involvement and musculoskeletal dysfunction. This study found that OMT provided significant pain relief and functional improvement, suggesting that similar mechanisms may underlie its effectiveness in treating foot drop [20].

Lastly, a study by Licciardone et al.(2020) found that OMT significantly improved outcomes in patients with chronic pain syndromes, supporting its role in comprehensive pain management strategies [15]. The study emphasized the importance of a holistic approach, integrating OMT with other therapeutic modalities to optimize patient outcomes.

Future directions

While this case demonstrates the effectiveness of OMT in providing significant symptomatic relief, further research is needed to understand the long-term benefits and potential for relapse. Developing standardized treatment protocols and conducting larger, controlled studies can help establish the role of OMT in managing foot drop and other neuromusculoskeletal conditions. The promising results observed in this case and supporting studies indicate that OMT could become integral to multidisciplinary treatment strategies for complex conditions like foot drop. This study underscores the need for further research and the potential for OMT to significantly impact the field of neuromusculoskeletal medicine.

## Conclusions

In the presented case, the patient suffered from a general diagnosis of foot drop. Upon osteopathic structural examination, she was assessed as having posterior fibular head somatic dysfunction, which was exerting pressure on the common fibular (peroneal) nerve. This pressure was relieved through OMT, which restored the corresponding structures to their correct anatomical positions and normal functions. The present case with treatment-resistant foot drop illustrates the diagnostic and therapeutic challenges inherent in managing complex neuromusculoskeletal conditions. Despite extensive evaluations and various treatment modalities, the underlying cause of her symptoms remains unclear to physicians not familiar with the biomechanical osteopathic approach. OMT provided complete resolutions of symptoms, suggesting a potential neuromusculoskeletal link and highlighting the utility of OMT as a therapeutic option.

This case also underscores the vital importance of a multidisciplinary approach in addressing complex medical conditions. Integrating osteopathic techniques with conventional treatments is not just beneficial, but necessary to optimize patient outcomes. Further investigation into the role of OMT in managing foot drop and similar conditions is warranted to establish its efficacy and develop comprehensive treatment protocols. If similar outcomes can be replicated in other patients suffering from foot drop, and especially foot drop that is resistant to other treatments, it may encourage broader acceptance of OMT among practicing clinicians, which in turn could significantly enhance healthcare outcomes. The positive outcomes observed in this case should be taken as a positive example for other healthcare providers seeking low-cost, non-invasive, first-line treatments for foot drop and similar neuromusculoskeletal issues.
